# Towards early software reliability prediction for computer forensic tools (case study)

**DOI:** 10.1186/s40064-016-2539-0

**Published:** 2016-06-22

**Authors:** Manar Abu Talib

**Affiliations:** Department of Computer Science, University of Sharjah, P.O. Box 27272, Sharjah, United Arab Emirates

**Keywords:** Reliability prediction, Computer forensic tool, Component-based, Markov model, COSMIC-FFP, ISO/IEC 19761:2003

## Abstract

Versatility, flexibility and robustness are essential requirements for software forensic tools. Researchers and practitioners need to put more effort into assessing this type of tool. A Markov model is a robust means for analyzing and anticipating the functioning of an advanced component based system. It is used, for instance, to analyze the reliability of the state machines of real time reactive systems. This research extends the architecture-based software reliability prediction model for computer forensic tools, which is based on Markov chains and COSMIC-FFP. Basically, every part of the computer forensic tool is linked to a discrete time Markov chain. If this can be done, then a probabilistic analysis by Markov chains can be performed to analyze the reliability of the components and of the whole tool. The purposes of the proposed reliability assessment method are to evaluate the tool’s reliability in the early phases of its development, to improve the reliability assessment process for large computer forensic tools over time, and to compare alternative tool designs. The reliability analysis can assist designers in choosing the most reliable topology for the components, which can maximize the reliability of the tool and meet the expected reliability level specified by the end-user. The approach of assessing component-based tool reliability in the COSMIC-FFP context is illustrated with the Forensic Toolkit Imager case study.

## Background

Kanellis et al. (2006) defined digital forensics as “the science of collecting evidence often used in a court of law to prosecute those who engage in digital activities that are deemed unlawful.” Reliable digital forensic techniques are therefore important for prevention, detection, and investigation of electronic crime.

As a new field, digital forensics requires computer forensic tools that ensure reliable results and meet the legal requirements acceptable in the courts. In the U.S., these tools should meet the four Daubert criteria: (1) testable and accurate, (2) peer reviewed, (3) accepted by the scientific community and (4) having acceptable error rates, which also requires intensive testing efforts (NIST 2001, 2005a, b).

According to Nelson et al. (2016) there are two types of computer forensic tools: hardware and software tools. Software forensic tools require versatility, flexibility and robustness. Many are closed-source, where only the vendor has access to the code, thereby making it more difficult to apply the Daubert criteria. This makes it imperative for researchers and practitioners to put more effort into assessing this type of tool (Abu Talib and Baggili 2016).

One of the main issues in developing computer forensic tools is the reliability of the tool components once combined. According to IEEE (1991), software reliability is “the probability of failure-free software operation for a specified period of time in a specified environment”. According to Ormandjieva et al. (2008), software failures are “primarily due to design faults. Repairs are made by modifying the design to make it robust against conditions that can trigger a failure. Software reliability has no wear-out phenomena or software errors occur without warning, and “old” code can exhibit a failure rate which increases as a function of errors introduced during upgrading. Moreover, external environmental conditions do not affect software reliability, while internal environmental conditions, such as insufficient memory or inappropriate clock speeds, do affect it.”

In this paper we improve the software reliability prediction model by extending the COSMIC-FFP method to component-based tools (ISO 14143-1 1988; ISO/IEC 19761 2003; Abran et al. 2009; Abu Talib 2007; Abu Talib et al. 2012). We model each component of the tool as a discrete time Markov chain and represent it as a finite state machine. The goals of the proposed method are: (1) determine the tool’s reliability in the first stages of implementation, (2) enhance the software reliability assessment mechanism for extensive computer forensic tools, and (3) examine substitute tool designs. The paper is organized as follows. “Work related to software reliability” section presents a brief survey of related accomplishments in software reliability models based on the theory of Markov chains, and discusses the major components of COSMIC-FFP together with a brief literature review of digital forensic tools assessment. “Proposed software reliability prediction methodology” section explains the methodology for predicting reliability in component-based tools; a case study in “Case study: Forensic Toolkit Imager” section illustrates the methodology. Finally, “Conclusion and future work” section summarizes the research results and identifies future research avenues.

## Work related to software reliability

### Markov model

Markov Processes have many applications in management and the environmental sciences. A prime illustration is the weather model (Wikipedia Encyclopedia), a powerful mathematical tool used by experts and engineers to investigate and anticipate the behavior of a complex component based system (Strook 2005; Trvedi 1975). In the last three decades several models using Markov chains have appeared in the literature. Nonetheless, existing models have the consistent issue of presenting the transition probability but no technique for deciding it (Lai-shun et al. 2011).

A Markov model analysis can produce a number of significant calculations that describe system performance such as system reliability, availability, mean time to failure (MTTF), or probability of being in a specific state at a specific time. Implementing a Markov model to predict software reliability has significant value for the following reasons:

- Environmental regulations fail to comply with component-based systems laws
- Within a given state, a component may randomly process a transition available in that state to move to another state.

Other research efforts in this direction include: (1) building a Markov chain-based software reliability usage model with UML (Lai-shun et al. 2011), (2) unifying the Markov model-based software reliability evaluation using failure data (Okamura and Dohi 2011), (3) using a Markov reliability model based on error classification (Jin et al. 2012), and (4) presenting software reliability test case design based on a Markov chain usage model (Wang et al. 2013).

### COSMIC-FFP measurement method

The Common Software Measurement International Consortium (COSMIC) developed the functional size measurement method (COSMIC-FFP), which was approved as international standard ISO 19761 (2003). Its design complies with all ISO provisions (ISO 14143-1) (ISO 14143-1 1998) related to functional size measurement systems. It was developed to cope with some key deficiencies associated with previous programs such as FPA, designed 30 years earlier when software systems had limited and basic specifications. COSMIC-FFP emphasizes the “user view” of functional requirements, and applies throughout the process life cycle, from the requirements stage through to completion and maintenance. Whenever the COSMIC-FFP method is used to measure software functional size the software functional processes and their triggering events must be determined. Data movement is the measurement unit, a base functional component that shifts one or more data attributes within the same data group. The four common data movement types are: Entry, Exit, Read and Write.

### Assessing and evaluating digital forensics tools

Without a clear strategy for empowering digital forensics research endeavors that expand upon each other, forensics research will fail to meet market expectations, digital forensic tools will become progressively out of date, and legal authorities, military and other clients of PC crime scene investigations will be not able depend on the results of digital forensic examinations (Garfinkel 2010). According to Flandrin et al. (2012) “Little research has been carried out on digital forensic tools evaluation and validation, which leaves investigators with few resources to assess their tools”.

Tool testing is important from an Information Technology (IT) perspective to ensure that software and hardware operate as expected. Tool testing programs have been initiated by various organizations. IEEE established standards for tool testing in 1993, while the International Organization for Standardization and the Electrotechnical Commission (ISO/IEC) established the General Requirements for the Competence of Testing and Calibration Laboratories (ISO/IEC 17025) in 1999 (General Testing Methodology 2007).

There is a high demand to evaluate computer forensic tools (Meyers and Rogers 2004). According to the National Institute of Standards and Technology (NIST), “there are three digital forensics projects currently providing resources for the digital investigator underway. These projects are supported by the U.S. Department of Justice’s National Institute of Justice (NIJ), federal, state, and local law enforcement, and the NIST Office of Law Enforcement Standards (OLES) to promote efficient and effective use of computer technology in the investigation of crimes involving computers.” Computer Forensic Tool Testing (CFTT) is one of these projects. NIST (Nelson et al. 2016; Lyle et al. 2008) performs the following steps in testing a tool:

1. Acquires the tool to be tested.
2. Reviews the tool documentation.
3. Selects relevant test cases depending on features supported by the tool.
4. Develops a test strategy.
5. Executes test cases.
6. Produces a test report.
7. Steering Committee reviews the test report.
8. Tool vendor reviews the test report.
9. NIJ posts the test report to the Web.

Moreover, there are ongoing assessment and evaluation efforts and research in this direction such as (1) NIST testing efforts (NIST 2005a, b; Nelson et al. 2016), (2) the first common evaluation scheme for forensic software, which is planned to be extensible and to bolster the benchmarking of forensics applications (Hildebrandt et al. 2011), (3) the validation and verification of computer forensics software tools (Guo et al. 2009) and (4) a high-level design for a second generation computer forensic analysis system based on metrics for measuring the efficacy and performance of computer forensic tools. The metrics include absolute and relative speed, reliability, accuracy, completeness, auditability and repeatability (Ayers 2009).

Our research work introduces new reliability analysis to assist designers in choosing the most reliable topology for the constituents, in order to expand the accuracy of the tool and comply with the desired reliability level required by the ultimate user. According to Ayers (Guo et al. 2009), “The tool must be designed and coded to provide a high level of assurance that analysis results will be correct and software operation free from error under all circumstances. Accuracy and reliability metrics must be 100 % in all validation tests.”

## Proposed software reliability prediction methodology

**Fig. 1:**
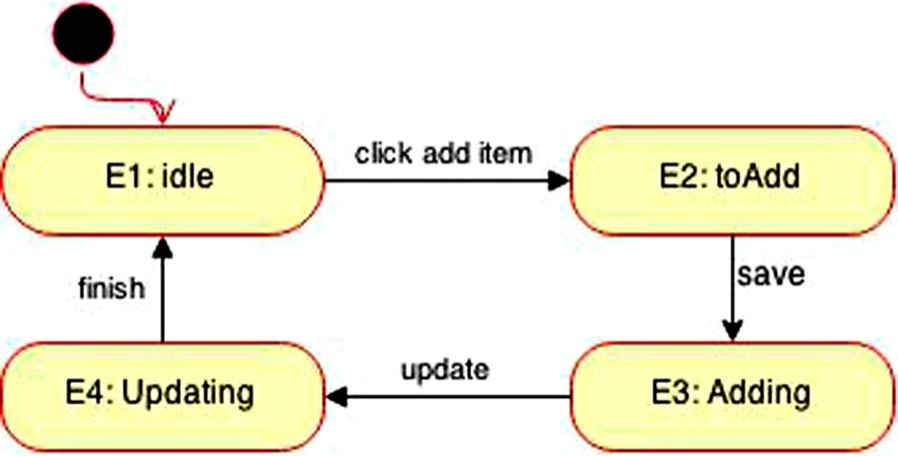
Evidence Item state machine diagram

A state-machine diagram is a UML 2.0 behavioral diagram (Rumbaugh et al. 2005; Gongzheng and Guangquan 2010; Booch et al. 1998) designed to illustrate the dynamic behavior of individual devices and describe the progress of the different states and the transitions involved. In Fig. 1, the state machine models the behavior of the Evidence Item in a Forensic Toolkit Imager case study, which has four states: one initial state, “idle”, “toAdd”, “Adding”, and “Updating”. When the “Add evidence item” option is clicked, it triggers the “Evidence Item” state to shift from the “idle” to the “toAdd” state.

**Fig. 2:**
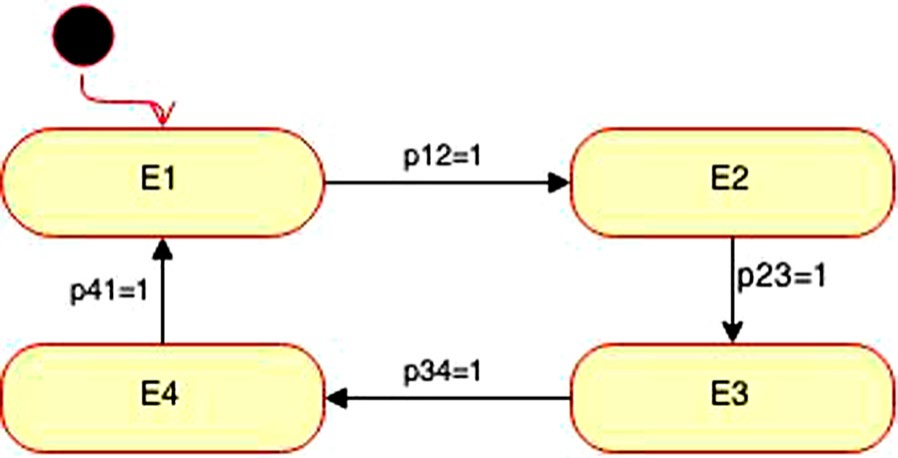
Evidence Item state diagram illustrating transition probabilities *p*_*i*j_

The purpose of a Markov model is to evaluate the accuracy of state machine programs (Ormandjieva 2002). Figure 2 illustrates the mapping of the Evidence Item object to a Markov model. Since there is only one event for each state, each event has a probability of 1, P12 represents the probability that the event will be triggered with the subsequent transition from state E1 to state E2.

Table 1 shows the transition matrix P for the Evidence Item Object that results from the state machine diagram where the *ij*th entry is *p*_*ij*_ and the entries in each row add up to 1. The prediction of reliability is derived from the steady state of the Markov model. The steady vector [wxyz] of the Evidence Item object is calculated based on the P matrix below:

$$\left| {\left[ {\text{wxyz}} \right]} \right|{\text{ P}} = \left| {\left[ {\text{wxyz}} \right]} \right| \to {\text{w }} = \, 0.25,{\text{ x }} = \, 0.25,{\text{ y }} = \, 0.25,{\text{ z }} = \, 0.25.$$

The steady vector value obtained is: [0.25, 0.25, 0.25, 0.25].

**Table 1:** Transition matrix ***P*** for Evidence Item object

	E1	E2	E3	E4
E1	0	1	0	0
E2	0	0	1	0
E3	0	0	0	1
E4	1	0	0	0

COSMIC-FFP and UML 2.0 state machine diagrams share the same concept as seen from a mapping of such concepts documented in Ormandjieva et al. (2008). Applying the COSMIC-FFP measurement method makes it possible for us to predict software reliability in the first stages of tool development. Our methodology for software reliability prediction for forensics tools is summarized as follows (Ormandjieva et al. 2008; Abu Talib 2007; Abu Talib et al. 2012):

1. State machine diagrams can result from the multiple interrelated sequence diagrams drawn using the COSMIC-FFP measurement method. This is shown in “Sequence diagrams from COSMIC-FFP” and “State machine diagrams from COSMIC-FFP” sections.
2. The probabilities of state transitions of an object due to events (environmental or related to that same object) are measured as displayed in “Markov model applications” section.
3. The product machine of the state machine objects pertaining to the same component results in an extended state machine outlining the behavior of the component. “Markov model applications” section illustrates this process.
4. The Markov model of a component is implemented in two stages. As an initial step, the Markov models are built for its objects. For the second stage, the Markov model for the entire component is built. This latter consists of synchronously interacting objects. “Markov model applications” section illustrates this process.

## Case study: Forensic Toolkit Imager

FTK Imager is a data preview and imaging tool that allows forensic investigators to assess electronic evidence. A Forensic Toolkit (FTK) helps to obtain, store, analyze, and provide computer evidence. To preserve the integrity of case evidence, forensic investigators do not work on the original files. Instead, they create an exact replica of the files and work on the image to ensure that the original files remain intact.

### Sequence diagrams from COSMIC-FFP

**Fig. 3:**
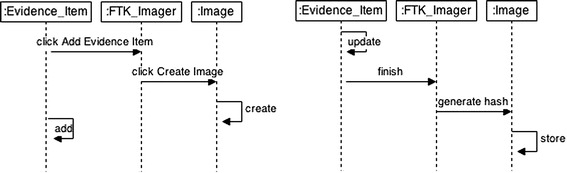
“Add Evidence Item” (part 1) & “Generate Hash” (part 2) sequence diagrams

A sequence diagram is a UML2.0 behavioral diagram (Rumbaugh et al. 2005; Gongzheng and Guangquan 2010; Booch et al. 1998), generally adopted for analysis and design that models the flow of logic within the system in a visual manner, enabling both documentation and validation of the user’s logic. In the RUP context (ISO/IEC 19761 2003; Abran et al. 2009), the functional processes used in COSMIC-FFP can explain the series of scenarios for the software. In the Forensic Toolkit Imager case study, for example, the first sequence diagram (Fig. 3, part 1) demonstrates that, when Add Evidence Item is clicked, the FTK Imager receives a message. The FTK Imager then instructs the Image to be created, and the Image is created. This process of adding Evidence Item and creating an image is referred to as a functional process, and is activated by clicking Add Evidence Item. Similarly, Fig. 3, part 2 is a scenario illustrating a sequence of events between the Evidence Item, FTK Imager and the Image. This scenario also incorporates a sequence of events within the tool (FTK Image in this case) to generate a hash image. Therefore, each functional process involves both its sub processes and its triggering events, which are sequences of events (or data movements).

### State machine diagrams from COSMIC-FFP

According to the COSMIC-FFP definitions given in ISO/IEC 19761 (2003) and Abran et al. (2009) and the sequence diagrams that derive from it, state machine diagrams can be obtained by applying these sequence diagrams. COSMIC-FFP measurements can be mapped to UML 2.0 state diagrams applying the technique proposed in Vasilache and Tanaka (2004), applied in Ormandjieva et al. (2008) (Abu Talib 2007; Abu Talib et al. 2012) and demonstrated by state machine diagrams from multiple interrelated scenarios (or sequence diagrams).

**Fig. 4:**
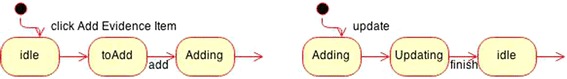
Initial state machine diagrams from Fig. 3

**Fig. 5:**
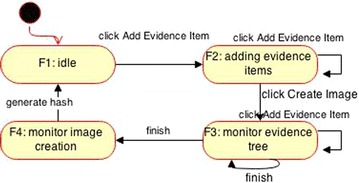
FTK Imager state machine diagram

**Fig. 6:**
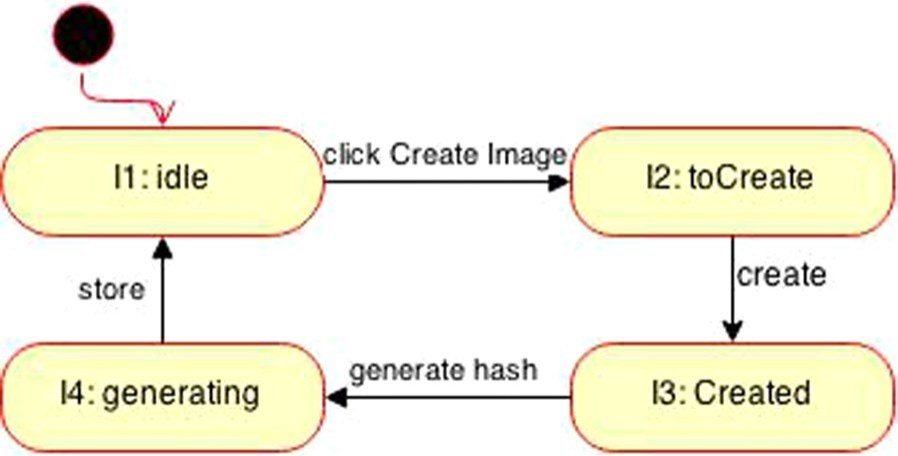
Image state machine diagram

The steps are summarized as follows (Ormandjieva et al. 2008; Abu Talib 2007; Abu Talib et al. 2012; Vasilache and Tanaka 2004):

Step 1: Draw sequence diagrams for all scenarios as illustrated in the previous section
Step 2: Draw a dependency diagram that shows the link between the series of scenarios (sequence diagrams) based on time dependencies between scenarios and dependencies related to their cause-effect and their generalization. In this case study we can say “Generate Hash” scenario depends on the “Add Evidence Item” scenario
Step 3: Create the state machines diagrams following the previous two steps. The sequence diagram in Fig. 3, part 1 has the following set of tuples = {(Evidence_Item, FTK_Imager, add_Evidence_Item), (Evidence_Item, Evidence_Item, add), (FTK_Imager, Image, click_Create_Image), (Image, Image, create)} while the sequence diagram in Fig. 3, part 2 has the following set of tuples = {(Evidence_Item, Evidence_Item, update), (Evidence Item, FTK_Imager, finish), (FTK_Imager, Image, generate hash), (Image, Image, store)}. Since three objects are involved in each scenario, three state machine diagrams can be derived as shown Fig. 4
Step 4: Adjust the final state machines and approve the compatibility between scenarios and state machines to ensure that the behavior of the final state machine diagrams reproduce the information contained in the scenarios (Figs. 5, 6)

### Markov model applications

The work reported here builds on our research results using the COSMIC-FFP method for testing purposes, by combining the functions measured by the COSMIC-FFP measurement procedure with a black box testing strategy (Abu Talib et al. 2005, 2006; Abran et al. 2004). This extends the COSMIC-FFP and reliability prediction model to the component-based tool context.

We can assume that each component in the tool is replaceable and functionally independent from the rest of the tool components. In order to predict the reliability of such a component, FTK Imager and Image objects are mapped to their corresponding Markov models as shown previously with the Evidence Item object. For example, the mapping of FTK Imager object to a Markov model assigns a probability of 1 for two events since only one event issues from F1 and F4 states, a probability of 1/2 for each of the two external events generating transitions from state F2, and 1/3 for each of the external three events issuing from F3. Table 2 illustrates the probability matrix for the map of the Image object to a Markov model, which is identical to the Evidence Item object mapping.

**Table 2:** Transition matrix ***P*** for FTK Imager and Image objects

	F_1_	F_2_	F_3_	F_4_		I_1_	I_2_	I_3_	I_4_
F_1_	0	1	0	0	I_1_	0	1	0	0
F_2_	0	^1/2^	^1/2^	0	I_2_	0	0	1	0
F_3_	0	0	^2/3^	^1/3^	I_3_	0	0	0	1
F_4_	1	0	0	0	I_4_	1	0	0	0

To ascertain the reliability of the component composed of Evidence Item, FTK Imager and Image objects, and calculate its level of uncertainty in the Markov model *H*, we applied the following formulas:

$$\begin{aligned} & Reliability \, \left( {Component} \right) \, = \, {\mathop{\varSigma}\limits_{i = 1,k}} H_{i} - \, H \\ & H \, = \, - \, {\mathop{\varSigma}\limits_{i}}\,v_{i}\,{\mathop{\varSigma}\limits_{j}}\,p_{ij}\,log_{2}\,(p_{ij} ) \\ \end{aligned}$$

where *H* stands for the level of uncertainty in a Markov chain corresponding to the whole component; *H*_*i*_ represents the level of uncertainty in a Markov chain corresponding to an object, *v* is a steady state distribution vector for the corresponding Markov chain, and *p*_*ij*_ are the transition probabilities in the extended state machines modeling the behaviors of the *i*th object (Ormandjieva et al. 2008; Abu Talib 2007; Abu Talib et al. 2012).

**Fig. 7:**
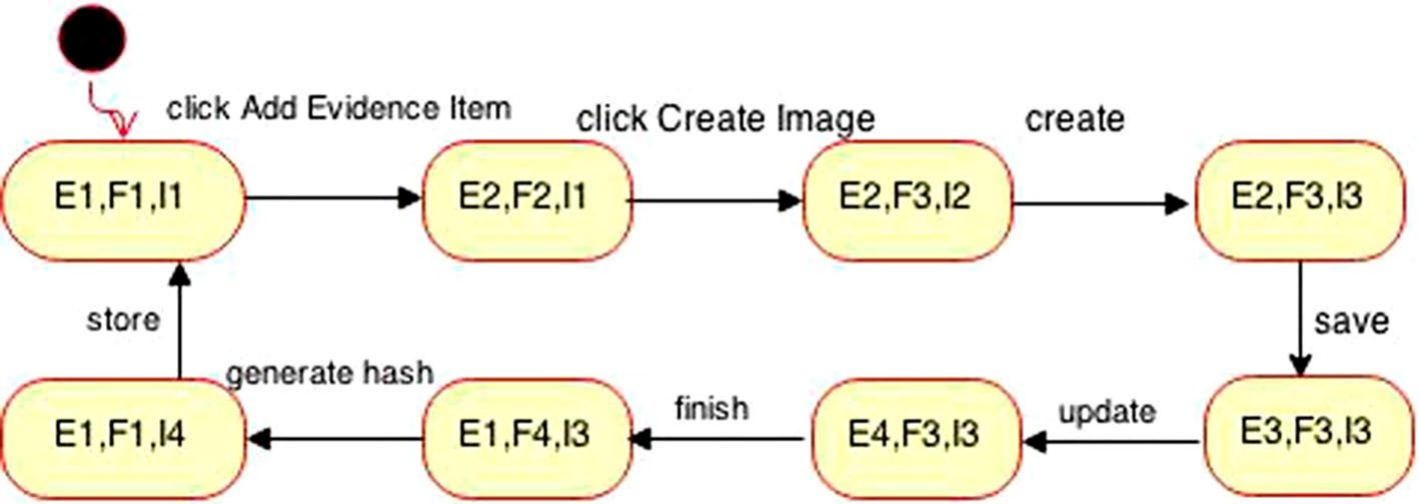
The synchronous product of Evidence Item, FTK Imager and Image objects as one component

The synchronous product of Evidence Item, FTK Imager and Image and its corresponding transition matrix P built to calculate *H*, are shown in Fig. 7 and Table 3.

**Table 3:** Transition matrix P of the component

	E_1_F_1_I_1_	E_2_F_2_I_1_	E_2_F_3_I_2_	E_2_F_3_I_3_	E_3_F_3_I_3_	E_4_F_3_I_3_	E_1_F_4_I_3_	E_1_F_1_I_4_
E_1_F_1_I_1_	0	1	0	0	0	0	0	0
E_2_F_2_I_1_	0	0	1	0	0	0	0	0
E_2_F_3_I_2_	0	0	0	1	0	0	0	0
E_2_F_3_I_3_	0	0	0	0	1	0	0	0
E_3_F_3_I_3_	0	0	0	0	0	1	0	0
E_4_F_3_I_3_	0	0	0	0	0	0	1	0
E_1_F_4_I_3_	0	0	0	0	0	0	0	1
E_1_F_1_I_4_	1	0	0	0	0	0	0	0

Table 4 shows the next steps in calculating H for each object within the above component.

**Table 4:**
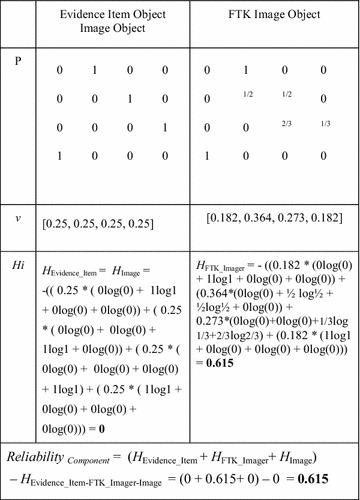
Calculating H for each object

We applied the same steps for other components in the same tool and compared the results. Major values of reliability measure indicate less uncertainty associated with the model, hence a higher level of software reliability. Adopting one evidence item implies less uncertainty in a Markov model for the FTK imager object and accordingly its behavior is not so complex that it necessitates generating additional sequences to describe it, while this is not the case for one FTK imager controlling two evidence items.

## Conclusion and future work

The advantage of the method reported in this paper derives from the ability to consider the measures of functionality early on where sequence diagrams are derived, as with COSMIC-FFP this makes it possible to take into account the uncertainty in the operational profile of forensic tools (i.e., the uncertainty of environmental events) as well as the uncertainty of failure of component behavior in forensic tools, based on:

- A component being recognized as a physical and substitutable part of the system which realizes, and conforms to, a set of interfaces (Jin et al. 2012); a component that is functionally detached from the rest of the components in a component-based system.
- Knowledge of the software architecture requirements (corresponds to reliability structures in reliability theory, see “Case study: Forensic Toolkit Imager” section).
- Evaluation of component reliability, in the context where a component is a group of interacting software objects the behaviors of which are modeled with state diagrams, and followed by application of the Markov model (see “Case study: Forensic Toolkit Imager” section). The probabilities of state transitions of an object generated by events (environmental or internal to the object) are measured as illustrated in “Case study: Forensic Toolkit Imager” section, where the environmental events are random and not regulated by system laws.

In the work reported here, the number of case studies was limited. Further work will explore how to approach such considerations as scalability and the processing of huge data. In addition, further specific templates are required for generating the set of scenarios and converting them into state diagrams.
